# An even drier future for the arid lands

**DOI:** 10.1073/pnas.2320840121

**Published:** 2023-12-29

**Authors:** Richard P. Allan, Hervé Douville

**Affiliations:** ^a^Department of Meteorology and National Centre for Earth Observation, University of Reading, Reading RG6 6BB, UK; ^b^Centre National de Recherches Météorologiques, Université de Toulouse, Météo-France, CNRS, Toulouse 31057, France

Recent heat extremes, wildfires, and droughts have afflicted multiple regions including parts of South America, the Mediterranean, and Middle East as well as the southwestern United States. These events underscore the vulnerability of societies to ongoing climate change as well as the precarious dependence of societies and ecosystems upon fresh water, the terrestrial lifeblood. A warming climate is projected to generate more intense wet as well as dry periods, and this is based on multiple lines of evidence (1–3) including computer simulations built with the most comprehensive representation of physics applied to the land, air, and sea (4). Long-term continental drying has previously been identified in terms of increasing aridity, reducing soil moisture, and also a decline in atmospheric relative humidity, a measure of how close to saturation the air is with gaseous water vapor (1). Greater warming over land than ocean is an expected consequence of global warming and implicated in continental relative humidity decline since the oceans are unable to supply enough moisture to maintain the levels of saturation over the hotter land (5). Building on previous findings of an overall underestimate in continental drying by climate models (6, 7) a new study in PNAS (8) exploits the most up-to-date observations and simulations to expound this discrepancy between models and observations: they identify a decline in water vapor over many arid and semi-arid continental regions that is contrary to expectations. This has implications for these already vulnerable regions which could experience an even drier future than predicted in response to global warming.

Basic physics dictates that the atmosphere’s thirst for water grows with warming, driving an increase in the gaseous form of water in the atmosphere. Through its ability as a potent greenhouse gas to absorb outgoing infrared radiation, as well as incoming sunlight, the increases in water vapor add to the temperature rise through a reenforcing feedback loop. A warmer, thirstier atmosphere can also more effectively sap moisture from the land or ocean in one region and transport this extra water into storm systems elsewhere, leading to intensification of heavy rainfall events as well as promoting more rapid onset of dry spells, with implications for worsening flooding as well as drought (1). It is therefore of utmost importance to establish fidelity in the processes determining water vapor changes in a warming climate. Although there are multiple lines of evidence to support the global increase in atmospheric moisture as the planet heats up (9, 10), the new research (8) finds compelling evidence of a deficiency in how climate models represent low-level moisture changes above many arid and partially arid regions across the globe.

Assessing simulated changes in surface water vapor requires scrutiny of a comprehensive set of observations from ground-based meteorological sites. Ensuring the fidelity of long-term surface humidity records is a particular challenge, requiring rigorous account for biases due, for example, to daytime solar heating of poorly ventilated instruments, as well as changes in observing practice and reporting (11). Presenting a convincing case that the measurements are robust, Simpson et al. (8) combine these records with a state-of-the-art global reanalysis (12) which uses multiple data sources to ensure that a complex physics-based global simulation remains as close as possible to reality.

**Fig. 1.**
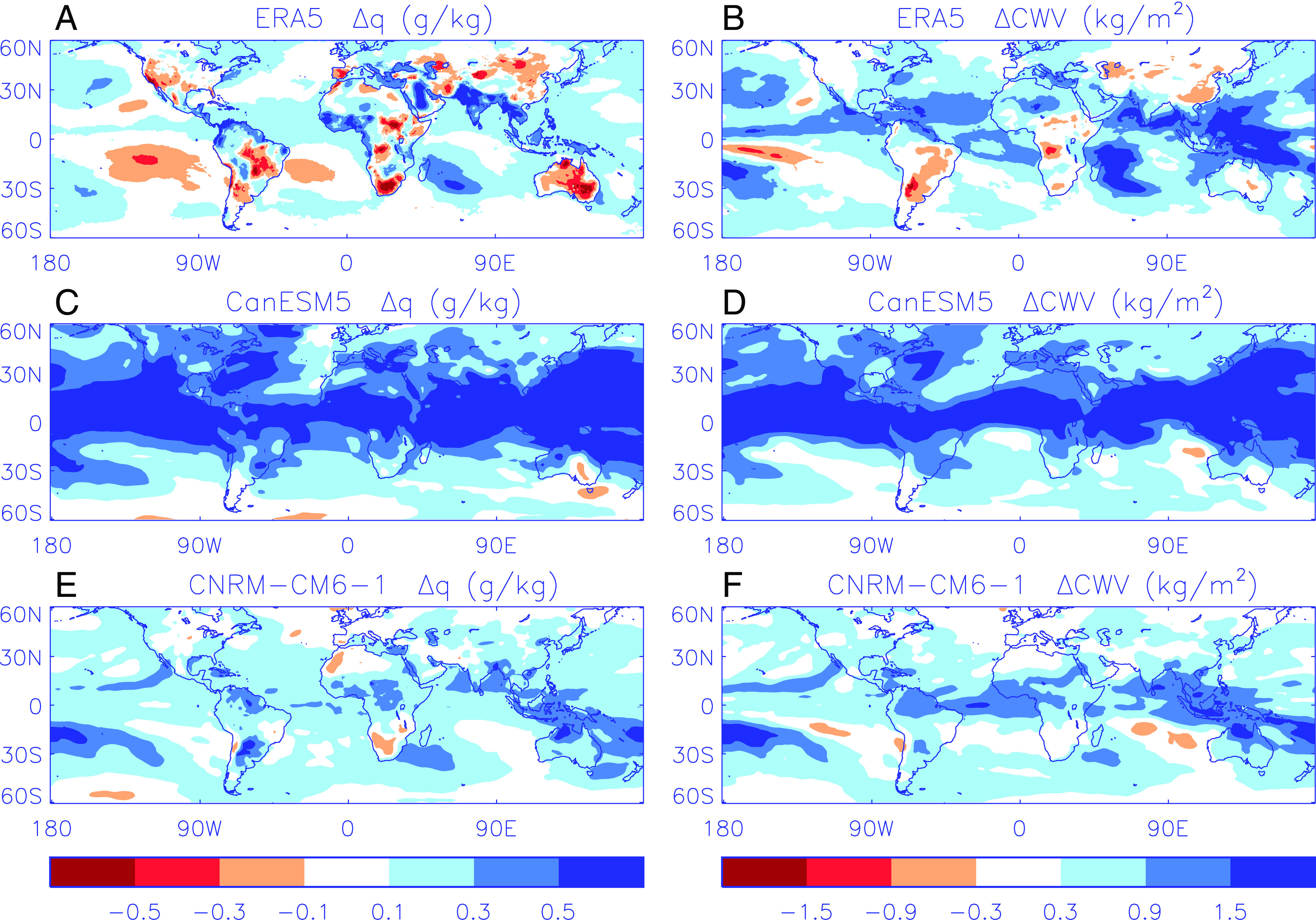
Changes in 2 m (near surface) specific humidity (q) and column integrated water vapor (CWV) from the 1988 to 2003 period to the 2004 to 2019 period from the ECWMF fifth generation reanalysis (ERA5; 12) (*A* and *B*) and two simulations (*C*–*F*) from the sixth phase of the Coupled Model Intercomparison Project (CMIP6; 4) combining historical and ssp2-4.5 (an intermediate greenhouse gas emissions scenario) experiments. The CanESM5 simulations produce a larger increase in moisture than the CNRM-CM6-1 simulation but neither are able to capture the magnitude or extent of decline in surface specific humidity (*A*) over arid and semi-arid regions such as the southwestern United States, central and southern South America, southern Africa, and Australia.

As an illustrative case study, the southwestern United States is highlighted as a key region exhibiting drying trends that is well endowed with reliable observations. The finding that water vapor amount has been declining rather than increasing here since the 1980s applies more broadly to drier climates globally (e.g., northern Argentina, southern Africa, parts of East Africa, western Mediterranean, central Asia, and eastern Australia) as well as to dry seasons in less arid climates. In contrast, climate models generally simulate increases in water vapor (Fig. 1) and even the most desiccating scenarios from the range of simulations considered fall short of the observed decline in atmospheric moisture over arid and semi-arid regions (8).

One suggested cause for the discrepancy between observed and simulated climate change is that the magnitude and pattern of global warming has diverged from the free-running simulations that generate their own climate variability through ocean and atmospheric fluctuations (13). Indeed, when the observed ocean warming pattern is fed into the model experiments, the discrepancy in continental drying diminishes, yet most simulations remain unable to capture the magnitude of drying, pointing to a more fundamental deficiency in model physics.

The drying signal is most acute at the lowest altitudes above the land surface (8, 9); this may provide a clue as to the causes of differences compared to model simulations of the historical period. Changing wind patterns, due to natural variations, or driven by longer-term climate change, can contribute to drying in some regions. However, accounting for possible changes in moisture-laden winds by selecting locations where precipitation has remained largely unchanged, Simpson et al. find over half of these dry areas exhibit reduced atmospheric moisture, in contrast to only about 10% or less of comparable regions in the simulations. This controlling for rainfall changes may suggest a more prominent role for evaporation, or the recycling of rainfall supplied by moisture transported by the winds (14, 15). Soil moisture has decreased in many regions over recent decades, particularly in the southern hemisphere (16), and these emerging trends in terrestrial water storage can be linked to multiple drivers including natural variability as well as human-caused climate change or direct water use (17, 18). However, more numerous dry days combined with an increased evaporative demand are likely to contribute to the observed subtropical drying through interaction between the land surface and the atmosphere (19).

Although the cause of the discrepancy in atmospheric humidity trends is unclear, Simpson et al. offer several plausible explanations: the ground may not have dried out as much as the real world; models may be leaking unrealistic amounts of moisture into the air; the partitioning of rainfall into runoff, soil moisture, or recycled back into the air through evaporation may be incorrect; or plants could be withholding their water as a result of their stomatal responses to rising levels of carbon dioxide (20), to an even greater extent than the models predict. Since the simulations are able to resolve the large-scale wind patterns and basic thermodynamic processes determining moisture supply, it seems more likely that processes operating at the land–atmosphere interface are involved in explaining the difference to the observed humidity changes. In particular, the fine detail characteristics of rainfall intermittency in space and time are not explicitly represented by the models and how precipitation is partitioned between runoff, infiltration, evaporation from the ground and transpiration from plants may be unrealistically simulated over drier climates. Land surface feedbacks involving soil moisture and vegetation are known to amplify continental drying (21) but multiple, interconnected processes need to be realistically modeled to obtain reliable simulations.

The implications of enhanced continental drying are substantial. Even drier arid zones will put further pressure on water resources and intensify extreme heat and wildfires. Beyond the subtropics, drier dry seasons in the tropics and mid-latitudes may also lead to damaging extreme events, as experienced across the globe in the early 2020s. A larger than simulated recent near-surface drying over arid regions may also imply a stronger than projected future continental warming (18). An even thirstier atmosphere can also lead to more rapid onset of drought that is already projected to intensify (22) while the greater swings between wet and dry (1–3) present a challenge for water resource planning which remains a neglected consideration in both mitigation and adaptation options (23). It is therefore crucial to fully understand why climate model simulations appear to underestimate drying over arid and partially arid regions. Although scrutiny of the observational records remains necessary to further confirm and characterize the continental drying, understanding differences with climate model simulations will require detailed and high-resolution regional modeling experiments to identify and correct deficient processes relating to rainfall characteristics and how this water is apportioned between the ground, vegetation, and the air. Ultimately, a potentially drier-than-anticipated future over arid regions will also require more extensive adaptation plans.

A new study by Simpson et al. exploits the most up to date observations and simulations to elucidate a discrepancy between models and observations: they identify a decline in water vapor over many arid and semi-arid continental regions that is contrary to expectations.
